# Predictors of resilience for people with spinal cord injury over two periods of COVID-19 social distancing restrictions: a 12-month longitudinal study using structural equation modelling

**DOI:** 10.1186/s12889-023-16238-x

**Published:** 2023-07-12

**Authors:** Ali Lakhani, Salvatore Dema, Josh Hose, Nazim Erdem, Dennis Wollersheim, Andrea Grindrod, Peter Grimbeek, Susan Charlifue

**Affiliations:** 1 The School of Psychology and Public Health, La Trobe University, 360 Collins St, Melbourne, VIC 3000 Australia; 2 The Hopkins Centre, Menzies Health Institute Queensland, Griffith University, Logan Campus, University Drive, Meadowbrook, QLD 4131 Australia; 3 Palliative Care Department, Eastern Health, 251 Mountain Highway, Wantirna, VIC 3152 Australia; 4 Austin Health - Royal Talbot Rehabilitation Centre, 1 Yarra Blvd, Kew, VIC 3101 Australia; 5 AQA Victoria, 416 Heidelberg Rd, Fairfield, VIC 3078 Australia; 6 Public Health Palliative Care Unit, School of Psychology and Public Health, La Trobe University, Kingsbury Drive, Bundoora, VIC 3086 Australia; 7 Upper Brookfield, Brisbane, QLD 4069 Australia; 8 Craig Hospital, Englewood, CO 80113 USA

**Keywords:** COVID-19, Disability, Resilience, Structural equation modelling, Longitudinal, Social distancing, Lockdown, Public Health

## Abstract

**Background:**

The novel coronavirus (COVID-19) pandemic is disproportionately impacting the health of people with disability. Resilience has remained an important health promoting characteristic during periods of social distancing restrictions. Factors promoting resilience for people with disability under the context of the pandemic remains poorly understood. Studies have yet to investigate evidence-based factors that promote resilience over multiple periods of restrictions for people with disability.

**Methods:**

A longitudinal study developed via a collaborative partnership between peer-support workers with lived experience of spinal cord injury (SCI) and university researchers was undertaken to fill knowledge gaps around factors promoting resilience for people with SCI during two periods of stringent social distancing restrictions within Victoria, Australia. Over 12-months, participants with SCI completed two surveys, towards the end of two lockdown periods. Evidence-based factors associated with resilience were measured. The Impact on Participation and Autonomy Questionnaire, the International SCI Quality of Life scale, and the 10-item Conor Davidson Resilience Scale, respectively measured autonomy and participation limitations, life satisfaction and psychological health, and resilience. A structural equation modelling (SEM) approach established factors directly and indirectly associated with resilience.

**Results:**

A model with excellent fit was produced. During two extended lockdowns over the 12-month period, increased family role limitations and favourable psychological health were respectively, negatively (Lockdown 1 [*n* = 127]: β = -.251, *p* < .01, Lockdown 2: β = -.400, *p* < .01) and positively (Lockdown 1: β = .601, *p* < .01, Lockdown 2 [*n* = 65]: β = .430, *p* < .01) associated with resilience. Indirect negative associations between resilience and increased outdoor autonomy limitations (Lockdown 1: β = -.195, *p* < .01, Lockdown 2: β = -.255, *p* < .01) and social life limitations (Lockdown 1: β = -.217, *p* < .01, Lockdown 2: β = -.142, *p* < .05) existed, and these relationships were moderated by psychological health.

**Conclusions:**

Psychological health, and participation and autonomy are determinants of resilience during periods of crisis. Health and social care providers and public health departments should prioritise programs promoting these domains, to counter the negative impact of social distancing.

## Introduction

Over a billion people globally live with disability, and as a result of chronic health conditions and demographic trends, this number is increasing (1). The COVID-19 pandemic is disproportionately impacting health outcomes of people with disability (1); compared to people without disability, people with disability face adverse social, economic, and health consequences (2–4). Resilience—defined as the ability to cope with and/or adjust to, adversity (5)—is associated with diverse health and wellbeing outcomes for people with disability, including mental, physical and social health outcomes (6), quality of life (7, 8), and activity continuation subsequent to a fall and/or injury (9). Increased resilience is aligned with an increased capacity to handle adversity (10). As those with heightened resilience are less likely to experience adverse health outcomes due to adversity, resilience is a health promoting attribute. For example, research has confirmed that resilience is a mediator between sleep problems and suicidal ideation (11), and increased resilience is associated with improved mental health (12, 13), reduced antidepressant use (14), and improved psychological health (10).

Resilience is especially important during the time of an emergency or crisis (15–17). Understandably, a growing body of research has investigated the impact of resilience on the health outcomes of people throughout the COVID-19 pandemic. Studies have confirmed that lower levels of resilience are associated with increased distress symptoms (18), and depression and anxiety (19–21). For people with disability, COVID-19 specific research has concluded that increased resilience protects mental health (22), is associated with reduced anxiety symptoms and improved quality of life (23), and supports the ability for people with disability to navigate and make decisions (24). COVID-19 disability specific research around resilience has a carer focus. In this respect, findings conclude that increased carer resilience, is inversely correlated with caregiver depression (25), whilst also associated with reduced stress related behaviour of children with neurodevelopmental disability (26). Limited research has investigated factors associated with and/or promoting resilience for people with disability during COVID-19. Research in this area has confirmed that for young people with disability, parenting self-efficacy (27), and for people with intellectual disability, day structure and routine and relationships (28) both promote resilience.

Research to date has been incredibly valuable and has improved our understanding surrounding the importance of resilience for people disability during periods of crisis. Specifically, current research has improved our understanding surrounding the impact of resilience on health and wellbeing outcomes for people with disability, and less so, surrounding factors promoting resilience for people with disability under the context of the pandemic. Most research to date has focused on caregiver perspectives, and as a result the perspectives of those with lived experience of disability are largely absent. Research to-date has been cross-sectional, collecting perspectives during a single period of social distancing restrictions. Many locations have experienced multiple waves of increased COVID-19 cases and social distancing restrictions. Still, longitudinal designs have yet to be employed within resilience specific COVID-19 research concerning people with disability. Given the protective nature of resilience, it is important to investigate the impact of public policies on resilience (19), and factors promoting resilience among people with disability during times of crisis. This is especially so moving forward from the pandemic (29) as resilience will remain a protective attribute which must be fostered among those who are characterised as vulnerable (30). This study aimed to fill knowledge gaps and investigated factors associated with resilience over multiple periods of increased COVID-19 cases and resulting social distancing restrictions, for people with spinal cord injury (SCI). It is expected that the findings from this research will inform policies and programs that aim to promote resilience during times of crisis (for example a pandemic).

### Study context

The state of Victoria, Australia experienced two periods (in 2020 and 2021) of heightened social distancing restrictions due to increased COVID-19 cases. During these periods, the restrictions were amongst the most stringent globally (31). Metropolitan Melbourne was under a strict lockdown from 30^th^ June 2020 (31, 32) until 28^th^ October 2020 (31, 33). From 30^th^ June to 2^nd^ August, Metropolitan Melbourne was under Stage 3 restrictions. A ‘state of disaster’ was declared on 2^nd^ August 2020, and during this period Stage 4 social distancing restrictions were enacted (34). These Stage 4 restrictions consisted of limited movement with a 5- km radius of the home, and an 8PM curfew. Residents could only leave home four essential reasons which consisted of exercise for up to 1-h a day, essential shopping, work and/or medical care. Mask wearing was mandatory within indoor and outdoor settings (except when undertaking rigorous exercise). Members from different households were unable to visit eachother. Stage 3 restrictions were comparable to Stage 4 restrictions as residents could only leave home for the four essential reasons, and there were no home visitors allowed (except for medical/care purpose). Stage 3 restrictions differed as residents were not required to remain within a 5-km radius of their home, and were not bound by a curfew (34). On 28^th^ October 2020, with eased cases, these stringent restrictions were lifted. However, with an increase in COVID-19 cases during 2021, social distancing measures were reenacted. These included short lockdowns in May, June and July, 2021 (28^th^ May to 10^th^ June, 16^th^ July to 27^th^ July) with an extended lockdown from 5^th^ August until the end of October 2021 [31, 35]. It is important to highlight that during the suggested periods, regional Victorian residents lived under Stage 3 restrictions, comparable to Stage 4 restrictions (see [36] for details). For details around the stringency of measures please consider the seminal Blavatnik School of Government Working Paper, where policy responses to COVID-19 across all Australian States and Territories have been documented [31]. Given the stringent level of restrictions within Victoria, Australia, it is an ideal location to investigate the factors which contribute to resilience during periods of lockdown or crisis.

### Study overview

A longitudinal study developed via a collaborative research partnership between peer-support workers with lived experience of SCI and university researchers in Australia, was undertaken. The study aimed to fill knowledge gaps around factors promoting resilience for people with mobile disability, people with SCI, during periods of increased social distancing restrictions and periods of isolation. In particular, it aimed to test for indirect and direct relationships between evidence-based factors associated with resilience for people with SCI. Factors had to meet two criteria and had to be (i) identified as significantly associated with resilience within a 2021 integrative review of 11 studies which aimed to establish the factors associated with resilience for people with SCI [37], and (ii) identified as important to consider by co-investigators with lived experience of SCI. The considered factors were psychosocial (social support, life satisfaction, and psychological health), and demographic (functional independence, employment status, age, gender and living location). Given relationships established within an integrated review [37], it was expected that being older, being employed, living in an urban location, having favourable psychological health, having increased social support, and having higher levels of functional independence would be associated with increased resilience. It was expected that considering the diverse factors contributing to resilience during a time of crisis, could inform public health policy and promotion activities.

## Methodology

This research was conducted in accordance with the Declaration of Helsinki, and received ethical approval from the La Trobe University Human Research Ethics Committee (Protocol ID: HEC20197). The study design and methods were developed in collaboration by the co-investigator team, consisting of peer-support workers with lived experience of SCI and university researchers. Specifically, the six-stage collaborative process model for university-community organization partnership was followed [38]. Within Step 1, through consultations between researchers and people with lived-experience of SCI, research aims and goals were established. During Step 2, researchers reviewed academic and grey literature for research in the area/s specified throughout consultations undertaken during Step 1. During Step 3, researchers presented collated information identified during Step 2, and participated in a consultation with people with lived experience of SCI, with the aim of confirming cross-sectional survey questions. Information presented during Step 3, was provided to people with lived-experience of SCI two-weeks prior to the session, to ensure adequate time for consideration. During Step 4, a consultation was held where the cross-sectional survey was presented to people with lived-experience of SCI, and feedback sought. (Similarly, information was provided two weeks prior to the consultation.) During Step 5, the revised cross-sectional survey accounting for feedback provided during Step 4, was presented to people with lived-experience of SCI, and any final feedback gathered and considered. During Step 6, the finalised survey was used.

### Study design

A longitudinal approach was utilised. An online survey was completed at three time-points. Participants completed the T1 survey, during September and October 2020. Participants responded to questions in light of their experience since the initiation of COVID-19 restrictions in March 2020. For a considerable portion of the reference period, participants were in stringent lockdown (experiencing either Stage 4 and Stage 3 restrictions during the latter three to four months). Participants completed a second survey between April and May 2021 (T2). Similarly, participants responded to questions in light of their experience over the previous six months, which coincided with a period where stringent social distancing restrictions had been lifted. Finally, between November and December 2021, participants completed a third survey (T3), where they provided their responses given their experience over the previous six months. During the majority (up to 4 months) of this six month period participants were living in stringent lockdown (experiencing either Stage 4 and Stage 3 restrictions). See Edwards, Barnes [31] for details around the extent of restrictions across the referenced periods. Given the study aims of establishing factors contributing to resilience during periods of social distancing restrictions, data from T1 and T3 have been utilised within this study (respectively referenced as Lockdown 1 and Lockdown 2 moving forward). This is the reasonable approach as it considers data collected over a 12-month period where participants provided responses given their experience whilst in two periods of heightened restrictions.

### Participants and recruitment

A convenience sampling approach was employed. Members and clients from a single health and social service organisation were recruited to participate in the study. The health and social service organisation provides advocacy services, and also assists people with lived experience organise their allied health support services. The health and social service organisation sent a personalised email to 1100 people with SCI. The information sheet about the study was attached to the email, and a weblink to complete the survey was included within the text of the email. The survey was completed via the REDCap online survey platform. Participants had to have an SCI and resided in Victoria to participate in this study. For participating, participants were entered into a draw to receive a $50 grocery stoe gift voucher (there were 10 vouchers available for each survey round). One hundred and twenty-seven people completed the Lockdown 1 survey and 65 of these participants completed the Lockdown 2 survey.

### Data collection

Participants completed the Impact on Participation and Autonomy Questionnaire (IPAQ) [39], which measures the level of difficulty that people with neurological disability, including SCI [40–43], have across the following domains: Autonomy Outdoors, Autonomy Indoors, Social Life and Relationships, Family role, and Work and Education. For the overall scale and each domain, a higher score is suggestive of having greater difficulty completing the domain. As previously reported [44] Lockdown 1 Cronbach’s alpha values were similar to Cardol, de Haan [40] suggesting the subscales are reliable. In relation to Lockdown 2 responses, Cronbach’s alpha values were similar to Lockdown 1 and Cardol, de Haan [40] suggesting the scales are reliable (see alpha in brackets): Autonomy Outdoors (0.83), Autonomy Indoors (0.92), Social Life and Relationships (0.87), Family Role (0.91), and Work and Education (0.89). Participants completed the International SCI Quality of Life (SCI-QOL) measure [45]. This measure is reliable for people with SCI [46], and includes three questions measuring satisfaction across, psychological health, physical health, and overall wellbeing. For each question participants are required to indicate how satisfied they are (0 [completely dissatisfied] to 10 [completely satisfied]). Cronbach’s alpha values for Lockdown 1 have been reported elsewhere [44] and were similar to findings by New, Tate [46]. Similar to Lockdown 1 and New, Tate [46] Cronbach’s alpha for Lockdown 2 indicated that the scale was reliable (0.87).

Two questions with dichotomous response options were utilised to establish whether participants received formal and informal peer-support (responses were coded as 1 [yes] or 0 [no]). Formal peer-support was described as support provided by someone with lived experience of SCI who was employed by a health and/or social service organisation (for example discussions with a peer-support worker). Informal peer-support was described as support provided by people with lived experience of SCI however not employed with a health and/or social service (for example conversations with someone with living with SCI). Additionally, two questions sought to establish how satisfied participants were with formal and informal support they received. Response options for these questions ranged from: 0 (completely dissatisfied) to 10 (completely satisfied).

The 10-Item Conor Davidson Resilience Scale [5] was used to measure resilience (a reliable measure for people with SCI [47]). The measure requires participants to indicate how much they agree with ten statements (scored from 1 [not true at all], to 5 [true nearly all the time]). A higher score is indicative of increased resilience. Cronbach’s alpha for Lockdown 1 and Lockdown 2 respectively were 0.92 and 0.87, mirroring reliability produced by Kuiper, van Leeuwen [47], and indicative of the scale being reliable.

### Data analysis

Measures aligning with psychosocial (social support, life satisfaction, and psychological health), and demographic factors (functional independence, employment status, age, gender and living location) were (i) identified as significantly associated with resilience throughout a 2021 integrative review of 11 studies [37], and (ii) identified as important to consider by co-investigators with lived experience of SCI. The factors and corresponding questions/measures have been included in Table 1 below.

**Table 1:** Factors and measures

Factors for Consideration	Measure Used
Social Support	Receipt of formal peer support (Y/N) Satisfaction with formal peer support Receipt of informal peer support (Y/N) Satisfaction with informal peer support Living with family and friends (Y/N) IPAQ—Social Support Subscale
Life Satisfaction	ISCIQOL—Overall Wellbeing Domain
Psychological Health	ISCIQOL—Psychological Health Domain
Functional Independence	IPAQ—Family Role Subscale IPAQ—Autonomy Indoors Subscale IPAQ—Autonomy Outdoors Subscale
Age	Age
Gender	Male
Employment Status	IPAQ—Work and Education Subscale Employed/Engaged in Activity (Y/N) Unemployed (Y/N) Retired (Y/N)
Living Location	Residing in Metropolitan Melbourne (Y/N)

Inferential analysis was progressed via a combination of IBM’s SPSS 26 and IBM’s AMOS 28. A three-stage approach was conducted. Initially, using Lockdown 1 data, Spearman’s rank-order correlation statistic was used to clarify whether a bivariate relationship between individual psychosocial and demographic factors and resilience existed. After, with IBM’s AMOS 28, a structural equation modelling (SEM) approach was undertaken to establish variables which were directly and/or indirectly associated with resilience. Variables which produced a significant bivariate relationship with resilience were included within the model. The SEM procedure was progressed as follows. Using Lockdown 1 data, a saturated model was developed where demographic and social support measures were included as level one variables, life satisfaction and psychological health measures included as level two variables, and resilience as the third-level outcome variable. The approach followed the modelling logic of Amato [48] and included demographic variables as the first block of variables. Social support was grouped with demographic factors as research involving people with SCI has confirmed that social support is associated with life satisfaction and psychosocial health [49], and further confirmed by study co-investigators with lived experience as important to group with demographic factors. Within the saturated model, all level one variables had a unidirectional link with level two variables and the single level three variable (resilience), and two level two variables had a unidirectional link to the single level three variable. All links which were non-significant or did not trend towards significance (*p* < 0.1) were removed, with the aim of identifying a parsimonious model which was intelligible. Once developed, the same model was applied to Lockdown 2 data.

Model fit was assessed  via the chi-square test for absolute fit (*p* > 0.05 indicative of the model fitting the data), and Comparative Fit Index (CFI) (≥ 0.95 indicative of an excellent fitting model) and Root Mean Square Error of Approximation (RMSEA) (≤ 0.06 indicative of an excellent fitting model) tests for relative fit.

### Findings

Demographic information for both groups has been included in Table 2 below. One hundred and twenty-seven completed the Lockdown 1 survey, and 65 people completed the Lockdown 2 survey.

**Table 2:** Demographic information

Domain	Lockdown 1 Frequency (Percent) /*Mean* (SD)	Lockdown 2 Frequency (Percent) / *Mean (SD)*
Sex		
Male	93 (73.2)	46 (70.8)
Female	32 (25.2)	19 (29.2)
Age (years)	*52.92 (13.88)*	*54.07 (12.85)*
Education		
Tertiary	91 (71.1)	44 (67.7)
Highschool or Under	32 (25.2)	19 (29.2)
Employment Status		
Employed or Engaged in Activity (working full-time or part-time, or volunteering)	65 (51.2)	32 (49.2)
Retired	24 (18.9)	14 (21.5)
Unemployed	36 (28.3)	17 (26.2)
Location		
Metropolitan	74 (58.3)	33 (50.8)
Regional	47 (37.0)	29 (44.6)
Home Ownership Status		
Home Owner	91 (71.1)	55 (84.6)
Renting or Social Housing	30 (23.6)	7 (10.8)
Household Composition		
Family and/or Friends	99 (78.0)	52 (80.0)
Alone	27 (21.3)	12 (18.5)
Primary Health Condition		
Paraplegia	57 (44.9)	32 (49.6)
Tetraplegia	61 (48.0)	29 (44.6)
Years with Condition	15.90 (12.92)	15.90 (13.07)

Descriptive statistics for scaled variables during both Lockdown 1 and Lockdown 2 periods are provided in Table 3. Spearman’s rank-order correlation statistic was used for Lockdown 1 data to establish variables to consider within SEMs. As clarified within Table 4, the following variables were significantly associated with resilience: Satisfaction with Formal Peer Support, Receiving Informal Peer Support, IPAQ—Social Support, SCI-QOL—Overall Wellbeing, SCI-QOL—Psychological Health, IPAQ—Work and Education, IPAQ—Family Role, IPAQ—Autonomy Indoors, and IPAQ—Autonomy Outdoors.

**Table 3:** Descriptive statistics for scaled variables

Domain	Lockdown 1 Frequency (Percent) /*Mean (SD)*	Lockdown 2 Frequency (Percent) / *Mean (SD)*
Functional Independence		
IPAQ – Autonomy Outdoors	*2.97 (.92)*	*2.44 (.88)*
IPAQ – Autonomy Indoors	*1.51 (.60)*	*1.54 (.67)*
IPAQ – Family role	*2.15 (.78)*	*2.05 (.83)*
Employment		
IPAQ – Work and Education	*2.58 (1.03)*	*2.44 (.92)*
Social Support		
IPAQ – Social Support	*2.03 (.68)*	*1.91 (.67)*
Receiving Formal Peer Support	75 (59.1)	33 (50.8)
Satisfaction with Formal Peer Support	*8.49 (1.80)*	*8.28 (2.02)*
Receiving Informal Peer Support	94 (74.0)	52 (80.0)
Satisfaction with Informal Peer Support	*8.48 (1.69)*	*7.68 (2.11)*
Life Satisfaction		
ISCIQOL – Overall Wellbeing Psychological Health	*6.57 (1.93)*	*7.02 (2.07)*
ISCIQOL – Psychological Health Resilience	*6.47 (2.42)*	*6.88 (1.97)*
Conor Davidson Resilience Scale	*3.87 (.67)*	*3.89 (.56)*

**Table 4:** Correlation coefficients

	95% Confidence Intervals			
Factor	Rho	*P*-Value	Lower	Upper
**Psychosocial Factors**				
**Social Support**				
Receiving Formal Peer Support	-0.102	0.272	-0.283	0.086
Satisfaction with Formal Peer Support	**0.411**	0.000	0.190	0.592
Receiving Informal Peer Support	**0.190**	0.039	0.004	0.363
Satisfaction with Informal Peer Support	0.205	0.058	-0.013	0.405
Living with Family or Friends	0.104	0.263	-0.084	0.286
IPAQ—Social Support	**-0.364**	0.000	-0.520	-0.184
**Life Satisfaction**				
ISCIQOL—Overall Wellbeing	**0.500**	0.000	0.347	0.628
**Psychological Health**				
ISCIQOL—Psychological Health	**0.622**	0.000	0.493	0.725
**Demographic Factors**				
Age	-0.034	0.723	-0.225	0.159
Male Gender	0.165	0.076	-0.023	0.341
Greater Melbourne	-0.006	0.946	-0.196	0.184
**Employment**				
IPAQ—Work and Education	**-0.353**	0.001	-0.530	-0.146
Employed/Engaged in Activity	0.074	0.428	-0.114	0.256
Unemployed	-0.116	0.212	-0.295	0.072
Retired	0.057	0.537	-0.130	0.241
**Functional Independence**				
IPAQ—Family Role	**-0.462**	0.000	-0.600	-0.297
IPAQ—Autonomy Indoors	**-0.392**	0.000	-0.541	-0.219
IPAQ—Autonomy Outdoors	**-0.360**	0.000	-0.514	-0.183

Lockdown 1 data underpinning the suggested variables were required to meet the following assumptions (as detailed by Aminu and Shariff [50]) necessary for SEM: not including a substantial percentage of missing responses, normality requirements, and having an absence of multicollinearity. There is no universal cut-off for the level of missing data which would inhibit inferential analysis [51], however, greater than 10% of missing data for a set variable can contribute to biased findings [52]. The following variables had greater than 10% missing data and were consequently excluded from SEM analysis (with percentage of missing data in brackets): IPAQ – Work and Education (27.6), and Satisfaction with Formal Peer Support (40.9). The level of missing data across both variables was likely because not everyone was working and/or receiving education and, not everyone engaged with formal peer-support. The remaining variables all had less than 7% missing data. Given both Lockdown 1 and Lockdown 2 data, the remaining variables were suitable for SEM analysis as they met normality requirements and multicollinearity was non-existent. All data fell within the acceptable skewness and kurtosis threshold [50, 53], respectively < 3 and < 10, and consequently met the criteria for normal distribution (a requirement for a SEM approach [53]). Furthermore, the remaining variables were not correlated at a level above a threshold of 0.9 [53], nor breaching tolerance (< 0.10) and Variance Inflation Factor (> 10) thresholds [53, 54], thus multicollinearity among independent variables was non-existent.

**Fig. 1:**
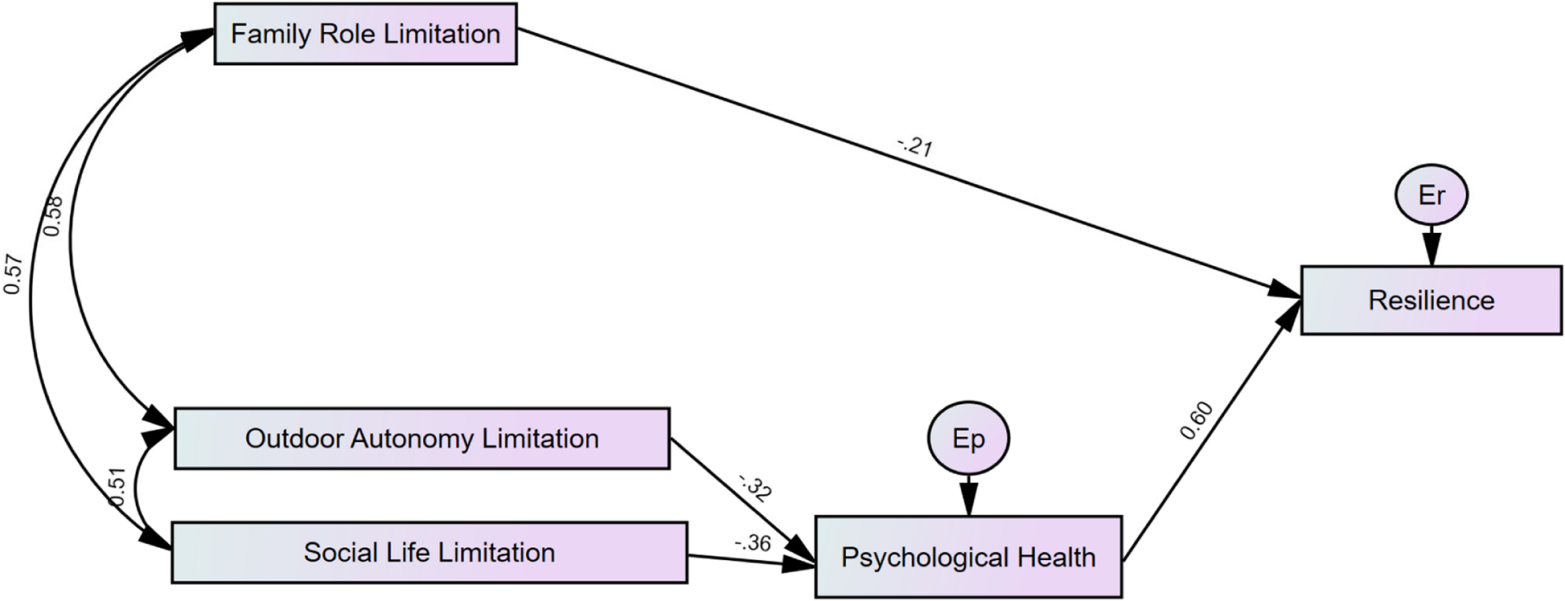
Structural equation model with Lockdown 1 Resilience as the outcome variable. Note: IPAQ-Family Role: Family Role Limitation, IPAQ-Outdoor Autonomy: Outdoor Autonomy Limitation, IPAQ-Social Role: Social Life Limitation, Er: Resilience Residual Error, Ep: Psychological Health Residual Error

**Fig. 2:**
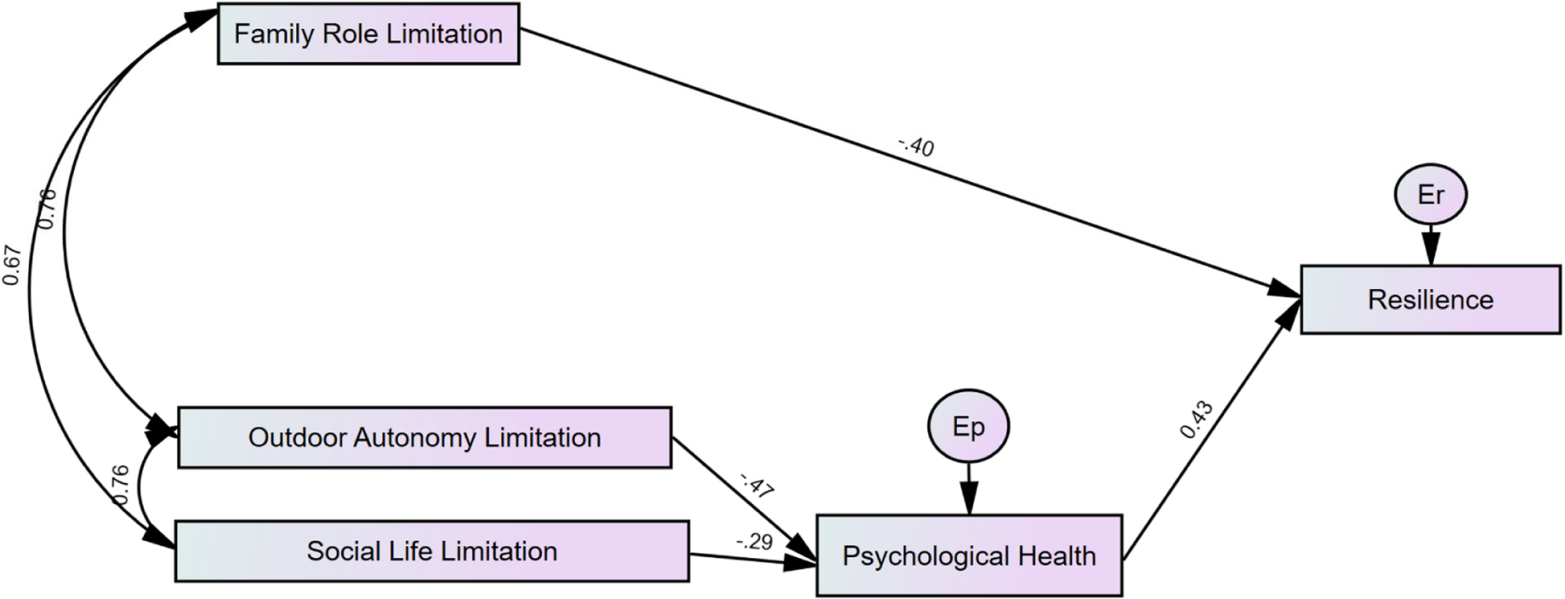
Structural equation model with Lockdown 2 Resilience as the outcome variable. Note: IPAQ-Family Role: Family Role Limitation, IPAQ-Outdoor Autonomy: Outdoor Autonomy Limitation, IPAQ-Social Role: Social Life Limitation, Er: Resilience Residual Error, Ep: Psychological Health Residual Error

The SEMs for Lockdown 1 and Lockdown 2 data are illustrated (with standardized coefficients) within Figs. 1 and 2 respectively. Both models exemplified excellent fit. Fit statistics for both models are as follows (with statistics in brackets): Lockdown 1 Resilience ([X^2^ [3] = 1.684, *p* = 0.640], CFI = 1.00, RMSEA = 0.000 [90% CI: 0.000, 0.120]), and Lockdown 2 Resilience ([X^2^ [3] = 2.672, *p* = 0.445], CFI = 1.000, RMSEA = 0.000 [90% CI: 0.000, 0.144]). Direct and indirect relationships between variables have been included in Table 5 and correlation coefficients are  provided in Table 6. For both Lockdown 1 and Lockdown 2 data, increased family role limitations and favourable psychological health were respectively negatively and positively associated with resilience. Increased outdoor autonomy and social life limitations were negatively associated with psychological health, and indirectly negatively associated with resilience. In summary, experiencing increased limitations inhibiting independence, is associated with lower levels of resilience, whilst having favourable psychological health, is associated with improved resilience. The relationships between increased limitations impacting outdoor autonomy and social engagement, and resilience, are moderated by psychological health.

**Table 5:** Structural equation modelling direct and indirect effect coefficients

**Effect**	**β**	**Confidence Interval (95%)**		***P*-value**
		**Lower**	**Upper**	
**Lockdown 1 Resilience**				
**Direct Relationships**				
IPAQ – Outdoor Autonomy –– > Psychological Health	-.324	-.454	-.156	< .01
IPAQ – Social Life –– > Psychological Health	-.362	-.520	-.175	< .01
IPAQ – Family Role –– > Resilience	-.251	-.357	-.078	< .01
Psychological Health –– > Resilience	.601	.477	.704	< .01
**Indirect Relationships**				
IPAQ -Outdoor Autonomy –– > Resilience	-.195	-.289	-.097	< .01
IPAQ – Social Life –– > Resilience	-.217	-.337	-.107	< .01
**Lockdown 2 Resilience**				
**Direct Relationships**				
IPAQ -Outdoor Autonomy –– > Psychological Health	-.472	-.693	-.244	< .01
IPAQ – Social Life –– > Psychological Health	-.362	-.527	-.007	.046
IPAQ – Family Role –– > Resilience	-.400	-.573	-.217	< .01
Psychological Health –– > Resilience	.430	.248	.570	< .01
**Indirect Relationships**				
IPAQ -Outdoor Autonomy –– > Resilience	-.255	-.444	-.106	< .01
IPAQ – Social Life –– > Resilience	-.142	-.329	-.013	< .05

**Table 6:** Structural equation modelling correlation coefficients

**Relationship**	**Estimate**	**95% Confidence Interval**		***P*-Value**
		**Lower**	**Upper**	
Lockdown 1 Resilience				
IPAQ -Outdoor Autonomy < –– > IPAQ – Social Life	.505	.353	.644	< .01
IPAQ -Outdoor Autonomy < –– > IPAQ – Family Role	.575	.440	.675	< .01
IPAQ – Family Role < –– > IPAQ – Social Life	.573	.416	.706	< .01
Lockdown 2 Resilience				
IPAQ -Outdoor Autonomy < –– > IPAQ – Social Life	.758	.603	.859	< .01
IPAQ -Outdoor Autonomy < –– > IPAQ – Family Role	.756	.625	.838	< .01
IPAQ – Family Role < –– > IPAQ – Social Life	.667	.441	.811	< .01

## Discussion

This is the first longitudinal study which utilised data from two COVID-19 lockdown periods, and tested for indirect and direct relationships between evidence-based factors associated with resilience for people with SCI. The findings from this study are robust as they involved identifying factors associated with resilience, and then confirming the appropriateness of those factors based on follow-up data. Initially a model of factors associated with resilience during a period of crisis (the first period of stringent social distancing restrictions in light of the COVID-19 pandemic) was developed. After modelled relationships were confirmed, based on data collected one year later during a second period of stringent restrictions. Thus, the factors indirectly (outdoor autonomy limitations and social life limitations) and directly (family role limitation and psychological health) associated with resilience for people with SCI during a time of crisis, identified within this study, should be considered reliable.

The factors were derived from a synthesis of the literature [37] and recognised as important to consider by co-investigators with lived experience of SCI. Findings confirm that psychological health, social health and functional independence contribute to resilience during stringent social distancing restrictions. When coupled with cross-sectional research focusing on resilience for people with disability and/or their carers during social distancing restrictions, and longitudinal research which considered data collected during social distancing restrictions and prior, the findings confirm factors important to consider for targeted health promotion interventions, and the nuanced relationship between psychosocial and demographic factors, and resilience.

The current study confirmed that psychosocial and functional independence variables (outdoor autonomy and family role limitations) were associated and contributed to a model which best fit resilience. Demographic variables were not significantly associated with resilience, and consequently, were not considered. In this respect, findings confirm contemporary longitudinal research in the area. In their study which aimed to assess the impact of the 2020 lockdown in Italy on the resilience of people living with multiple sclerosis Sbragia, Colombo [55], tested for a relationship between psychological health, functional independence, and demographic factors collected prior to the lockdown, and resilience as measured during the lockdown. Favourable psychological health and greater functional independence were associated with higher resilience, whilst demographic factors were not. In combination, it appears as though for people with disability, including people with SCI, functional independence and psychological health are resilience promoting characteristics during periods of social distancing, and perhaps in general times of crisis.

As to a large degree, psychological health and functional independence are modifiable determinants of resilience, it is worthwhile for health and social care providers, communities and public health departments, to consider interventions and programs which can promote psychological health and functional independence in general, especially during periods of crisis. In relation to promoting psychological health, some favourable interventions are web based, thus have particular value during periods of social distancing restrictions, or during times where physical contact is not possible (for example, natural disasters including extreme weather events). Some examples include engaging with: natural environments delivered via virtual reality [56], web-based guided cognitive behavioural therapy [57], and web-based health coaching [58]. In relation to functional independence, providing allied health support [59, 60], removing environmental barriers [61] (physical and social at home and community levels) and implementing assistive technologies [62] (at home and community levels), may promote functional independence outcomes. Again, such initiatives can be particularly valuable during periods of social isolation or crisis.

The findings confirm that social engagement has an indirect effect on resilience, moderated via psychological health. Increased social life limitations were negatively associated with psychological health and resilience. These longitudinal findings are confirmed by cross-sectional research investigating factors associated with resilience among young people during the initiation of social distancing restrictions in Canada [27]. In their cross-sectional study, Yusuf, Wright [27] found that increased parent support in accessing school was associated with resilience among young people (demographic variables were not). In combination, the findings suggest that programs which promote social participation can contribute to increased resilience during times of crisis. Given our understanding of factors associated with social participation for people with a SCI, programs which assist in health condition management [42] and contribute peer-support engagement [63] may work to reduce social life limitations.

Throughout multiple periods of social distancing restrictions, factors associated with resilience remained consistent. Study findings confirmed that direct and indirect relationships between factors associated with resilience identified during Lockdown 1, were comparable to Lockdown 2 (based on β coefficients and *p*-values); the notable exception being the relationship between social life limitations and psychological health, which was significant during Lockdown 1, while trending towards significance during Lockdown 2. Furthermore, correlation coefficients and *p*-values during both periods were comparable. These findings confirm that factors promoting resilience among people with SCI during consecutive periods of crisis (for example lockdowns), may remain the same. Thus, for people with SCI, targeted efforts to promote resilience during an initial period of crisis may still be worthwhile during a subsequent period.

It is important to consider study limitations as they impact the implications of findings. Data underpinning this study were derived during two periods of social distancing restrictions, and at the time, these restrictions were amongst the most stringent globally, and certainly within Australia [31]. Consequently, the factors contributing to resilience and aligned recommendations are likely relevant for regions which have experienced relatively stringent restrictions, and in the future, regions which experience extreme isolation because of crisis. The current study first involved, developing a parsimonious intelligible model based on Lockdown 1 data (*n* = 127), and applying this same model and calculating parameter estimates using Lockdown 2 data (*n* = 65). There is no universally understood method to calculate the sample size required for a structural equation model [64–66]. The 10:1 cases (samples) to parameter ratio has been tested and identified as acceptable [64, 66]. The model utilised within the current study, includes seven parameters, thus the analysis based on Lockdown 1 data is well powered (*n* = 127 vs the sample of 70 required), while the analysis based on Lockdown 2 data is near to well powered (*n* = 65 vs the sample of 70 required). Given the restricted and distinct sample (people with spinal cord injury), and unprecedented scenario (experience of multiple stringent lockdowns, which at the time, were amongst the most stringent globally, in light of a global pandemic), estimates derived from Lockdown 2 data, should be considered. This aligns with the perspective provided by Barrett [67], suggesting that SEM sample size concessions should be made for small samples, when the population is restricted. Given the unique scenario that the study investigated, it is expected that confirmation of these findings would be extremely difficult, thus the current findings are valuable.

## Conclusions

Compared to people without disability, people with disability, including SCI, can experience adverse participation and wellbeing outcomes. Such differences can be heightened during times of crisis, necessitating targeted support to promote health. Resilience is a determinant of health, and programs which aim to promote resilience are exceptionally important. Ongoing efforts to improve determinants of resilience, including functional independence, psychological health and social participation, are necessary. It is expected that such efforts will be especially beneficial during times of crisis and worthwhile to consider as it may reduce the amplified health and wellbeing gap between people with and without disability that exists during times of crisis.

## Data Availability

The data underpinning the current study is not publicly available. Identifiable information was collected, and participants consented to the raw data being accessible to the research team only. If someone wishes to request data from this study, please contact Dr Ali Lakhani (a.lakhani@latrobe.edu.au).
